# Scurvy: A Neglected Cause of Muscle Pain and Weakness in a Child With Picky Eating Behavior

**DOI:** 10.7759/cureus.16289

**Published:** 2021-07-09

**Authors:** Ren Yi Kow, Nik Alyani Nik Abdul Adel, Ardilla Hanim Abdul Razak, Chooi Leng Low, Mohd Shukrimi Awang

**Affiliations:** 1 Department of Orthopaedics, Traumatology & Rehabilitation, International Islamic University Malaysia, Kuantan, MYS; 2 Department of Radiology, International Islamic University Malaysia, Kuantan, MYS

**Keywords:** scurvy, vitamin c, ascorbic acid, paediatric orthopedics, muscle pain, weakness, eating behavior

## Abstract

Ascorbic acid (vitamin C) is an essential micronutrient that the human's body cannot synthesize endogenously. Scurvy, a disease of ascorbic acid deficiency, can manifest in a myriad of presentations. Due to its rarity in the modern world, scurvy is considered as a disease of the past. We present a paediatric case of scurvy with musculoskeletal manifestations as a result of picky eating behavior.

We report a previously healthy nine-year-old boy who presented with unexplained progressive bilateral lower limb generalized weakness and pain for two months. All initial biochemical and radiological investigations were unremarkable. Upon further history taking, he had severe picky eating behavior which raised the suspicion of scurvy. The diagnosis was confirmed with a serum ascorbic acid test. After ascorbic acid supplementation, his symptoms resolved immediately. Further food behavioral modification counselling to his family members helped to change his diet in a lasting way. As a result, he had no recurrence of symptoms.

This case highlights the importance of having a high index of suspicion for an uncommon disease and emphasizes the need for a detailed dietary history upon patient’s presentation.

## Introduction

Scurvy, a disease due to deficiency of vitamin C (ascorbic acid), has already been described in ancient times [1-3]. First described in Ebers Papyrus in 1500 BC, this disease was common in sailors who had inadequate intake of vitamin C during their voyage [3]. Sir James Lind was the first who discovered lemons and oranges to be the treatment for scurvy [1,3]. Nevertheless, it was not until 1931 that ascorbic acid was found to be the active ingredient in citrus fruits for the prevention and treatment of scurvy [3].

In the modern era, the incidence of scurvy is very rare. Even in developing countries, the easy access to tropical fruits made scurvy a nearly obsolete disease [1-3]. Patients with scurvy usually have nutritional insufficiency (e.g., extreme poverty) or as a sequela of avoidant-restrictive food intake disorder [4]. Clinical presentations of scurvy are varied, ranging from non-specific symptoms such as fatigue, aching pain and irritability to bleeding gums and impaired wound healing [2-4]. In paediatric patients, they commonly present with musculoskeletal dysfunction as the first symptom, with or without cutaneous manifestations [5,6]. Nevertheless, due to its rarity, scurvy is often not within the list of differential diagnoses of treating physicians. We report a rare case of scurvy causing unexplained muscle pain and weakness in a child to highlight this rare but easily treatable disease.

## Case presentation

A nine-year-old boy with no known medical illness presented with progressive bilateral lower limb generalized weakness and pain for two months. The pain and weakness started insidiously at the left lower limb and subsequently affecting both of his lower limbs. The pain and weakness were non-specific, affecting the hips, thighs, knees and calves. The pain was persistent and aggravated by movements and manipulation. It was partially relieved by rest. Initially, he was limping, however, symptoms worsened and he subsequently became bed-bound. He was not able to score the pain but he claimed the pain had progressively worsened. Both of his upper limbs were asymptomatic and were normal on neurological examination. There was no history of trauma or fall prior to the development of his symptoms. There was no sign of infection such as fever or localized erythema.

His antenatal and postnatal history were unremarkable. He had an episode of febrile fits at the age of one but there was no recurrence since then. His immunization was up-to-age. He was initially treated at two district hospitals but to no avail and he was then referred to our institution for further investigations and management. Further history revealed that he was having intermittent gum bleeding and selective eating with strong aversion towards fruits and vegetables since he was three-year-old. 

**Figure 1:**
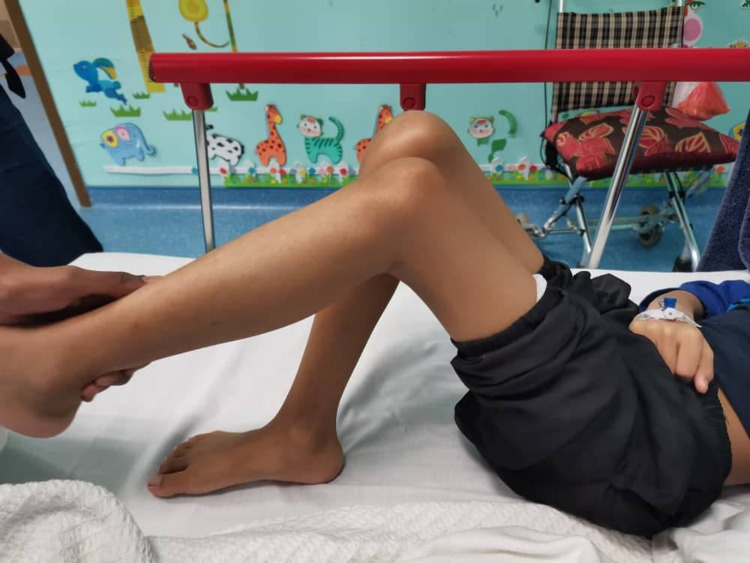
The patient's lower limbs were in fixed flexion position and any attempt to straighten the leg would induce pain.

**Figure 2:**
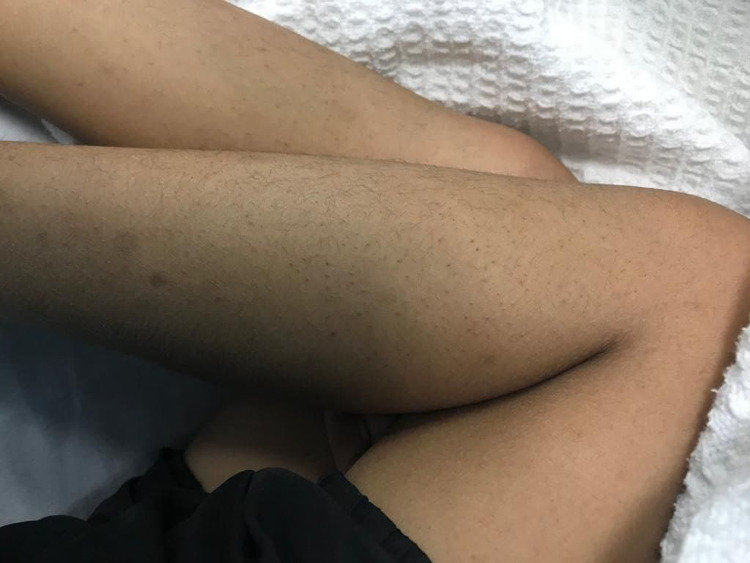
Petechiae present at both legs.

Generally, his body weight was slightly lower than the 5th centile but his body mass index was between the 5th and 10th centile. Clinically, there was no swelling or wound on his lower limbs. His lower limbs were in a flexed position (Figure 1) and there was presence of petechiae (Figure 2). A full examination of the lower limbs was not completed as he was fretful and refused to co-operate due to pain.

Basic biochemical examinations did not reveal any abnormality except for a slightly elevated erythrocyte sedimentation rate (ESR). Plain radiographs of the lower limbs were normal. Similarly, magnetic resonance imaging of whole spine and bilateral lower limbs did not reveal any abnormality. In the early stage of presentation, we encountered diagnostic dilemma as the cause of the clinical problem was obscured by normal investigation results. We did consider muscle biopsy and manipulation of the knee joint under general anaesthesia until we obtained the history of his picky eating behavior and subsequently worked towards the diagnosis of scurvy. As a result, his serum ascorbic acid level was found to be significantly low (<0.1 mg/dL). The investigation results are summarized in Table 1.

**Table 1:** Summary of the biochemical and radiological investigations done for this patient.

Investigation	Result (reference range)
White cell count	6.4 x 10^9^/L
Creatine kinase (CK)	58 U/L (2-177)
C-reactive protein (CRP)	1.67 umol/L
Erythrocyte sedimentation rate (ESR)	56 mm/hr
Lactate dehydrogenase (LDH)	225 U/L (110-295)
Complement C3	1.8g/hr (0.9-1.8)
Complement C4	0.53g/hr (0.1-0.4)
Blood culture and sensitivity (C&S)	No growth
Anti-streptolysin O	Negative
Rheumatoid factor	Positive 1:4
Mycoplasma lgM & lgG	Negative
Urinalysis	Normal
Plain radiographs	Normal
Magnetic resonance imaging (MRI) spine	Normal
Magnetic resonance imaging (MRI) bilateral lower limb	Normal
Serum ascorbic acid	<0.1mg/dL (0.4-2.0)

He was subsequently started on vitamin C supplementation (100 mg tablet three times a day) and an immediate clinical improvement was observed. His pain resolved after 3 days of oral ascorbic acid supplementation and he was able to walk five days after the initiation of ascorbic acid supplementation. Parents were counselled on the cause of the disease and advice to provide a nutritionally complete diet for the patient was given. After desensitization to curb his eating disorder, he started to drink fruit juice but still maintained the rejection for leafy vegetables. At one-year follow-up, there was no recurrence of gum bleeding, muscle pain and weakness. 

## Discussion

Around 61 million years ago, human ancestors had lost the ability to synthesize ascorbic acid endogenously [7]. Inactivation of L-gulonolactone oxidase (GLO) gene results in blockage of ascorbate synthesis from glucose, making ascorbic acid an essential micronutrient that must be attained from diet [7]. Fortunately, there are plenty of easily accessible ascorbic acid-rich food such as fruits, potatoes and tomatoes in the world [2,4]. Even in a developing country like Malaysia, scurvy is a rare disease due to the abundance of tropical fruits which are rich in ascorbic acid.

Ascorbic acid is pivotal in multiple biochemical pathways in a human body. Ascorbic acid is a cofactor for prolyl and lysyl hydroxylase which is crucial in the hydroxylation step of collagen synthesis [3]. In patients with scurvy, lack of ascorbic acid results in tissue and capillary fragility. These patients usually present with cutaneous manifestations such as easy bruising, ecchymosis and gum bleeding [3,5]. Besides that, ascorbic acid also helps in iron absorption, without which one may present with anemia [5]. Paediatric patients often suffer from subtle musculoskeletal manifestations [8]. Like our patient, they can present with refusal to weight-bear or non-specific pain and swelling involving the extremities [8]. Pain and swelling can be attributed to synovial blood vessel injury, microfractures, subperiosteal hemorrhage and haemarthrosis [5]. In patients with prolonged deficiency of ascorbic acid, osteoporosis may develop as lack of ascorbic acid promotes bone resorption [5]. 

To diagnose scurvy in a patient, a serum ascorbic acid will be sufficient. Nevertheless, the treating physician should exclude other more sinister causes such as hematological malignancy, infections, auto-immune diseases, and trauma that may immobilize a child. Although biochemical tests such as full blood count, erythrocyte sedimentation rate (ESR), C-reactive protein (CRP) and radio-imaging may be helpful in reaching the diagnosis, they are of no substitute for a thorough history taking and detailed clinical examination of the patient. In younger children, special precautions must be exercised so that a non-accidental injury will not be missed. Through this case, we would like to highlight the importance of taking a detailed dietary history in a paediatric patient who presents with unexplained pain. In our patient, despite knowing that he has picky eating behavior, the diagnosis of scurvy is not in our list of consideration until we have exhausted a range of diagnostic tests due to its rarity in Malaysia. 

The recommended daily allowance (RDA) of ascorbic acid is 60 mg/day and the requirement raises with increased metabolic demand of the body [5]. Thus far, there is no standardization of the treatment dose of ascorbic supplementation in patients with scurvy, but previous reports show that consuming 300 mg to 1 gram of vitamin C per day have been successful in resolving symptoms within five days. In our patient, his symptoms subsided within 3 days after oral ascorbic acid supplementation of 300 mg/day and he was able to regain his normal daily activities in five days.

The root cause of scurvy is picky eating in this patient. Although picky eating behavior is common in children, children with severe food selectivity are at risk of developing scurvy and rickets [3,4,9]. Studies found that picky eating is a result of hereditary factors and child-parent interaction [9]. In consistent with previous studies, our patient developed picky eating behavior at the age of three years [9]. Although this patient has been having picky eating behavior since young, he did not develop any symptoms of scurvy that require medical attention until the age of nine-year-old. We postulate that the patient might be having intermittent mild symptoms that were previously undetected and the symptoms resolved upon ingestion of ascorbic acid-rich foods. In patients with picky eating behavior that has jeopardized their health, interventions must be administered. In this case, we encourage the following: (1) graded and repeated exposure to unfamiliar food and fruits; (2) promote appetite by limiting junk food and substitute it with fruits; (3) having positive approach, and avoid pressuring the child to eat; (4) parental modelling of eating fruits and vegetables; (5) associate food experience with family bonding; (6) be consistent and focus on long term habit of eating healthy food. At one year after the first presentation, he did not have any recurrence of symptoms. 

## Conclusions

This case highlights scurvy as a rare cause of muscle pain and weakness in a paediatric patient. A high level of suspicion on this uncommon disease should be practiced whenever a paediatric patient presents with unexplained muscle pain and weakness. A detailed dietary history should be acquired for all the paediatric patients with similar complaint so that this easily treatable disease will not be missed.
